# Financial incentives for quitting smoking in pregnancy: Are they cost‐effective?

**DOI:** 10.1111/add.16176

**Published:** 2023-03-15

**Authors:** Nicola McMeekin, Lesley Sinclair, Lyn Robinson‐Smith, Alex Mitchell, Linda Bauld, David M. Tappin, Kathleen A. Boyd

**Affiliations:** ^1^ Health Economics and Health Technology Assessment, Institute of Health and Wellbeing University of Glasgow Glasgow G12 8RZ UK; ^2^ York Trials Unit, Department of Health Sciences, Faculty of Science University of York YO10 5DD York UK; ^3^ Usher Institute and SPECTRUM Consortium University of Edinburgh Edinburgh EH8 9AG UK; ^4^ Child Health, School of Medicine, Dentistry and Nursing University of Glasgow Glasgow UK

**Keywords:** Child health, cost‐effectiveness analysis, economic evaluation, financial incentives, pregnancy, smoking cessation

## Abstract

**Aims:**

To evaluate whether adding financial incentives to usual care is cost‐effective in encouraging pregnant women to quit tobacco smoking, compared with usual care alone.

**Design:**

Cost‐effectiveness analysis (CEA) and cost–utility analysis (CUA) from a health‐care provider's perspective, embedded in the Smoking Cessation in Pregnancy Incentives Trial (CPIT III). Long‐term analyses were conducted from the same perspective, using an existing Markov model over a life‐time horizon.

**Setting:**

Seven maternity smoking cessation sites in Scotland, England and Northern Ireland in the United Kingdom.

**Participants:**

In the short‐term analysis, CPIT III participants were assessed: women 16 years or older, self‐reporting as smokers, fewer than 24 weeks pregnant and English‐speaking (*n* = 944). The same population was used for the life‐time analysis, plus their infants.

**Measurements:**

Costs included financial incentive vouchers and postage, cessation support and nicotine replacement therapy and neonatal stays. The outcome measure was a biochemically verified quit rate for the CEA and quality‐adjusted life‐years (QALYs) for CUA. Costs are presented in 2020 GBP sterling (£). Data for the life‐time analysis came from the trial and were combined with data from published literature embedded in the model, reporting incremental cost per quitter and QALY. A 3.5% discount rate was applied.

**Findings:**

The short‐term incremental cost per quitter was £4400 and cost per QALY was £150 000. Results of sensitivity analyses confirmed these results. The long‐term analysis combined costs and outcomes for mother and infants; results showed a cost saving of £37 [95% confidence interval (CI]) = £35–106] and increase in QALYs of 0.171 (95% CI = 0.124–0.229). These findings indicate that, over a life‐time, financial incentives are cost‐saving and improve health outcomes.

**Conclusions:**

In the United Kingdom, offering up to £400 financial incentives, in addition to usual care, to support pregnant women to stop smoking appears to be highly cost‐effective over a life‐time for mother and infants.

## INTRODUCTION

Tobacco smoking is the principal cause of preventable deaths globally, linked to 8 million deaths annually world‐wide and over 91 000 in the United Kingdom [1]. Tobacco smoking prevalence during pregnancy is 1.7% globally [2]; however, in the United Kingdom it is higher. In 2020/2021, it was 9.6% in England [3] and 13% in Scotland [4]. Smoking during pregnancy is linked to low birth weight and increased risk of premature birth [5]. After pregnancy, passive smoking is linked to an increased risk of sudden infant death syndrome, lower respiratory diseases, asthma and impaired lung function in infants [5]. Children living in households with smokers are also 90% more likely to take up smoking than children living in non‐smoking households [6].

In addition to the burden on health to mother and infant, the economic burden of smoking during pregnancy is estimated to be more than £23 million annually in the United Kingdom [7].

As 14% of women are smokers at conception in England [8], pregnancy poses a good opportunity to quit smoking, improving the health of the mother and infant and reducing pressure on health‐care budgets.

All pregnant women in the United Kingdom are offered National Health Service (NHS) stop smoking support (SSS) and nicotine replacement therapy (NRT) to stop smoking, but few use this service and set a quit date (11%), and even fewer (3.5%) remain abstinent 4 weeks after their quit date [9]. Financial incentives have been shown to be effective in supporting women to quit tobacco during pregnancy; a recent Cochrane Review combined the results of nine trials to estimate a relative risk of 2.38 [95% confidence interval (95% CI) = 1.54–3.69], favourable to financial incentives [10]. One of these nine trials was a single‐site Phase II trial in Scotland, Smoking Cessation in Pregnancy Incentives Trial II (CPIT II), which found that offering a maximum of £400 financial incentives resulted in higher quit rates; this research was carried out by the CPIT trial team [11]. CPIT II estimated a life‐time incremental cost per quality adjusted life‐years (QALY) of less than £500, which is considered highly cost‐effective when compared to the National Institute of Health and Care Excellence (NICE) willingness‐to‐pay threshold of £20 000 [12, 13]. Following the CPIT II trial, a financial incentive scheme was introduced in NHS Greater Glasgow and Clyde (NHSGG&C); this was found to be effective in improving quit rates at 4 and 12 weeks post‐quit, and the incremental cost per quitter was less than £550 for both 4 and 12 weeks post‐quit date [14]. While this is encouraging, additional evidence is needed from multi‐site research with a longer follow‐up period providing information on relapse post‐birth [15].

More recently, CPIT III evaluated the effectiveness of offering up to £400 financial incentives to support pregnant women to stop smoking [16]. This paper reports the economic evaluation of CPIT III, exploring whether financial incentives are cost‐effective in encouraging pregnant women to stop smoking.

## METHODS

### CPIT III overview

CPIT III was a pragmatic, multi‐centre, randomized controlled trial which assessed the effectiveness and cost‐effectiveness of offering financial incentives in addition to usual care, compared to usual care only, to improve the smoking quit rate in pregnant women. Service delivery varied between sites, but usual care was typically SSS plus NRT for 12 weeks.

Participant inclusion criteria included pregnant women self‐reporting as smokers, 16 years or older, fewer than 24 weeks pregnant and English‐speaking. Participants were recruited from seven sites across England, Scotland and Northern Ireland between February 2018 and April 2020.

The trial primary outcome was quit rate at late pregnancy (34–38 weeks’ gestation) with those self‐reporting as quit confirmed as abstinent by biochemical verification. Participants had further follow‐up to 6‐months post‐partum to establish the biochemically verified sustained quit rate.

### Economic evaluation

Two time horizons were considered: a short‐term within‐trial and life‐time trial, both taking an NHS perspective. Cost‐effectiveness was reported as cost per quitter and cost per QALY.

This research follows best practice for methods [17, 18] and reporting of economic evaluations [19]. The health economics analysis plan is available elsewhere [20] (there is a deviation from protocol, as mean costs and outcomes are presented with standard errors, not standard deviations, as stated in the protocol [20]).

### Treatment arms

The intervention arm consisted of financial incentives worth up to £400 in shopping vouchers plus usual care. Participants received a £50 voucher for engaging with stop smoking services (SSS) and setting a quit date. Participants who were carbon monoxide (CO)‐verified as quit at 4 and 12 weeks post‐quit date received £50 and £100 vouchers if they met the criteria for the previous voucher. Participants received a final £200 voucher if CO‐verified quit at late pregnancy; participants could still receive this voucher if they had not met criteria for previous shopping vouchers. Due to the COVID‐19 pandemic, an amendment was made for participants reporting smoking status after 16 March 2020: self‐reporting for quit at 4 and 12 weeks, and participants self‐reporting as quit at late pregnancy received the final voucher if they provided a saliva sample. The control arm was usual care only.

### Within‐trial analysis

The population for the within‐trial analysis was that of the CPIT III trial. The time horizon for this analysis was from recruitment into the trial to birth; as this was less than 1 year, discounting was not applied to costs or outcomes.

#### Resource use

Resource use categories include issued vouchers, postage for vouchers, SSS, NRT and neonatal costs. The trial data management system recorded when vouchers were sent to participants and a standard postal charge was applied to each voucher. During the trial, 87 vouchers were re‐sent, 59 of which required a postage charge; this charge was also captured.

Individual participant details of SSS received and NRT prescribed were collected for five sites using NHS routinely collected data and bespoke Excel spreadsheets. For the remaining two sites, site‐specific typical SSS and NRT use for an individual (established using expert opinion) was applied to participants reporting NRT use in the trial database.

Neonatal care stays were not collected during the trial, so gestational age (preterm status) at birth was used as a proxy. Preterm was classified by severity as defined by the World Health Organization as follows: ‘extremely preterm’ (< 28 weeks gestation), ‘very preterm’ (28–32 weeks gestation) and ‘moderate to late preterm’ (32–37 weeks gestation) [21]. Length of stay was applied to each class of preterm birth: 93 days for extremely preterm, 44 days for very preterm and 13 days for moderate to late preterm [7].

##### Unit costs

Unit costs were obtained from routine sources (Table 1), including British National Formulary [22], Personal Social Service Research Unit [23], NHS National Cost Collection [24] and the CPIT trial team. Costs are reported for the price year 2020 and expressed in pounds sterling (GBP£).

**TABLE 1:** Unit costs.

Variable	Unit cost	Source
Voucher engagement and setting quit date	£50	CPIT III Trial
Voucher 4‐week CO verified quit	£50	
Voucher 12‐week CO verified quit	£100	
Voucher late‐pregnancy CO verified quit	£200	
Voucher postage	£2.92 per voucher	
Resent voucher postage	£2.05 per voucher	
Sensitivity analysis postage	£6 per voucher	
Smoke‐free adviser/midwife per hour (grades 5/6 depending on site)	£39/£49	PSSRU 2019/2020
Neonatal costs per day (moderately preterm)	£536.45	NHS National Cost Collection version 2 2019/2020, health‐care resource group XA05Z Neonatal Critical Care, normal care
Neonatal costs per day (very preterm)	£709.16	NHS National Cost Collection version 2 2019/2020, health‐care resource group XA03Z Neonatal Critical Care, special care
Neonatal costs per day (extremely preterm)	£1707.5	NHS National Cost Collection version 2 2019/2020, health‐care resource group XA01Z Neonatal Critical Care, intensive care

Abbreviations: CO, carbon monoxide; CPIT, Smoking Cessation in Pregnancy Incentives Trial; NHS, National Health Service; SSRU, Personal Social Services Research Unit.

Unit costs were combined with resource use data to estimate a mean cost per participant in each trial arm. Generalized linear model (GLM) regression analysis was conducted to estimate the cost difference between arms, adjusting for site, baseline age, number of years of smoking and primary outcome collection pre‐ or post‐16 March 2020 [25].

#### Outcomes

Two outcomes were used: late‐pregnancy quit rate and QALYs. The timing of late pregnancy was used as a proxy for birth due to the difficulties of collecting data at birth. A QALY combines health‐related quality of life and quantity (length) of life. Quality of life was measured using the EQ‐5D‐5L questionnaire [26], completed by participants at baseline, late pregnancy and up to 6 months post‐partum. Responses were converted to health utilities using the UK value set [27] and cross‐walk mapping, as recommended by NICE [28, 29]. Quantity of life was measured by the length of time a participant remained in the trial. A standard area‐under‐the‐curve approach was used to calculate QALYs, with changes in utilities between follow‐up points treated as linear [30, 31]. GLM regression analysis was conducted to estimate the difference between arms, adjusting for site, baseline age and utilities, gestational age at booking and primary outcome collection pre‐ or post‐16 March 2020 [30].

#### Analysis of costs and effects

Mean costs and outcomes are presented with standard errors (SEs) for each arm and differences between arms are presented with 95% CI. Cost per late‐pregnancy quitter and cost per QALY incremental cost‐effectiveness ratios (ICERs) are presented, and the latter is compared to the UK NICE willingness‐to‐pay threshold of £20 000 to assess cost‐effectiveness [13].

#### Uncertainty

Uncertainty in the results was explored with non‐parametric bootstrapping using 1000 iterations [32, 33]. The bootstrapped results were plotted on a cost‐effectiveness plane. A cost‐effectiveness acceptability curve (CEAC) for the QALY outcome is presented with varying willingness‐to‐pay thresholds to explore cost‐effectiveness at different thresholds.

#### Missing data

Participants with missing quitter outcome data, both for the primary outcome and late‐pregnancy quit rate, were assumed to be smokers, as per Russell standard best practice [34].

Missing cost and QALY data were assessed as missing at random (explained by the variables miscarriage, stillbirth and trial site) [35]. Multiple imputation using chained equations was used to replace missing data at a disaggregated level, following best practice recommendations [35].

#### Sensitivity analyses

Ten sensitivity analyses were conducted to explore the effects on the results of altering inputs, these were (1) including miscarriage as a covariate in the GLM regression, (2) using self‐reported smoking status at late pregnancy, (3) adjusting for possible gaming (where participants self‐report quit, they are biochemically verified quit but a residual blood sample fails to confirm quit status) based on evidence from trial (incentives, two of 18, 11%; control, two of 10, 20%), (4) adjusting for possible gaming using incentives arm only (conservative estimate), (5) 6‐month post‐partum quit rate, (6) 6‐month post‐partum QALYs (from EQ‐5D‐5L responses), (7) complete case analysis, (8) trial mean neonatal costs (same in both arms) to test impact on results if difference in preterm births observed in trial was down to chance, (9) including postage per voucher at £6 and (10) including postage per voucher at £0. The latter three analyses were not pre‐specified, but added *post hoc*. The postage analyses were conducted to explore potential implementation scenarios. Analysis was undertaken using Stata version 17 (StataCorp. 2021; Stata Statistical Software, release 17; College Station, TX, USA).

### Life‐time analysis

In the short term we would not expect quit rates to translate into immediate health gains, so long‐term analysis is needed to reflect the impacts of improved quit rates. The life‐time analysis was conducted to capture all relevant costs and benefits of these impacts as per best practice recommendations [17]. These results will be more relevant for decision‐making than the within‐trial results, which are primarily used for input into the life‐time analysis. This analysis utilized a published model developed for assessing the cost‐effectiveness of interventions for smoking cessation in pregnant women [7]. The discount rate used was 3.5% for costs and QALYs, as recommended by NICE [13].

#### Model structure

**FIGURE 1:**
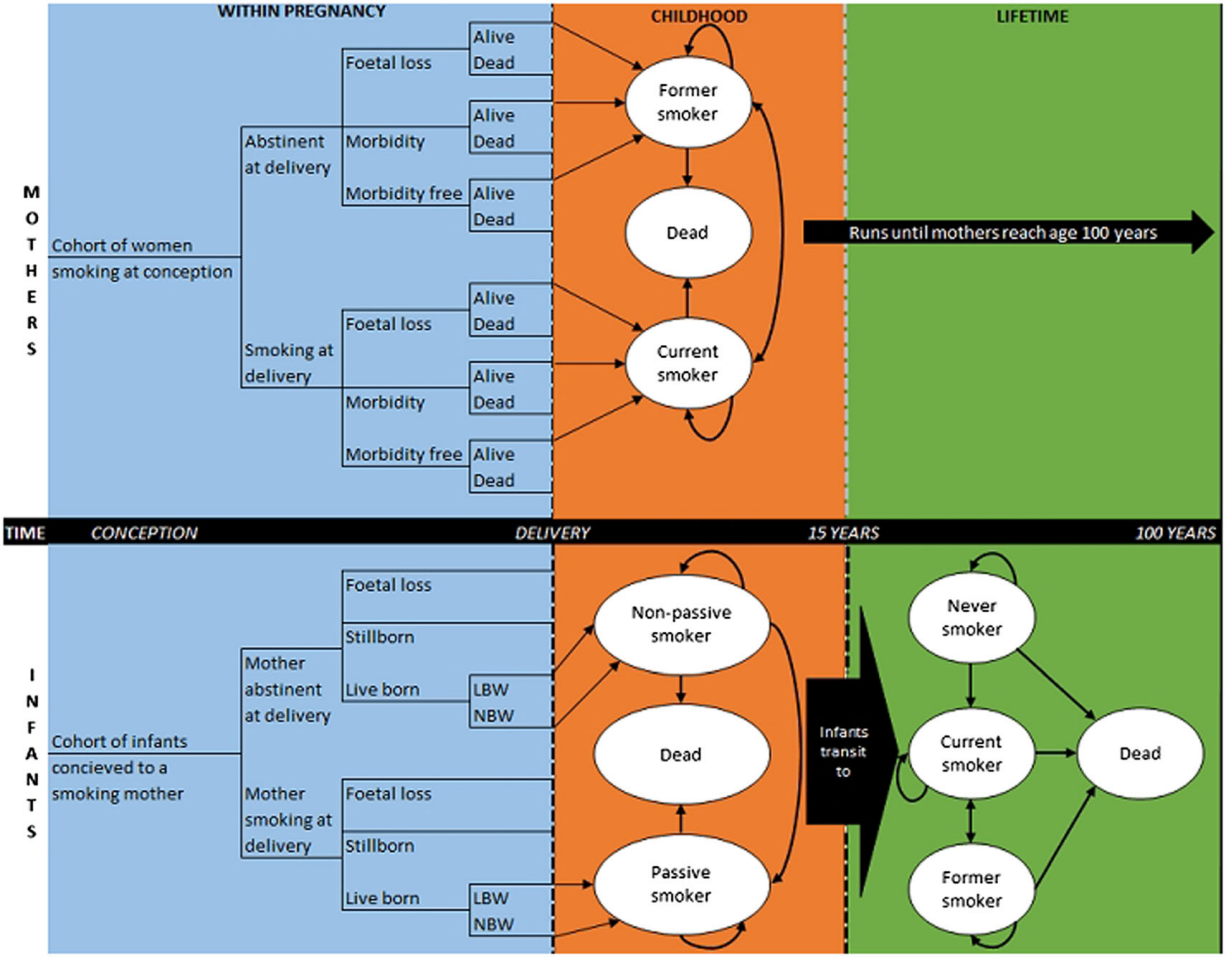
Model structure (Jones *et al.* [36]).

The Economics of Smoking in Pregnancy (ESIP) model combines decision trees and Markov models to estimate an incremental cost per QALY for mother and infant during a life‐time (up to age 100 years) (Figure 1). Further details regarding the model are available elsewhere [7, 36]. In summary, the model combines two cohorts (mothers and infants) and is split into three sections: pregnancy, childhood (birth to 15 years) and life‐time (100 years maximum). In each section, smoking‐related morbidity and mortality is applied to both cohorts to account for the effects of smoking. Costs and QALYs are accumulated in all sections for both cohorts.

#### Parameters

Treatment costs (intervention and control arms) and quit rates from the CPIT III trial were input into the model for base‐case and sensitivity analyses (Table 2). Further details of existing parameters in the model are available elsewhere [7]. Similar to the short‐term analysis, late pregnancy was used as a proxy for birth.

**TABLE 2:** ESIP model input parameters.

Parameter	Input value	Source
Base case		
Cohort size	944	CPIT III trial data
Year or pregnancy	2019	CPIT III trial data
Age of mother	28	Mean age of participants in CPIT III (age at baseline)
Discount rate for costs/QALYs	3.5%	NICE reference case (13)
Quit rates at late pregnancy	Financial incentives 0.268 (SE = 0.020) Control 0.123 (SE = 0.015)	CPIT III trial data (quit rate at late pregnancy)
Cost of intervention and comparator	Financial incentives £262 (SE = 11.7) Control £91 (SE = 6.40)	CPIT III trial data
Willingness‐to‐pay threshold	£20 000	NICE reference case (13)
Sensitivity analyses		
(1a) Gaming: quit rates	Financial incentives 0.239 (SE = 0.020) Control 0.098 (SE = 0.015)	CPIT III trial data (quit rate at late pregnancy—adjusted for within‐trial gaming, incentive reduce by 11%, control reduce by 20%)
(1b) Gaming: quit rates	Financial incentives 0.239 (0.020) Control 0.109 (0.015)	CPIT III trial data (quit rate at late pregnancy—adjusted for within‐trial gaming, reduce incentives and control quit rates by 11)
(2) Self‐report quit rate (late pregnancy)	Financial incentives arm 0.359 (SE = 0.022) Control arm 0.185 (SE = 0.018)	CPIT III trial data (self‐reported quit rate, with missing smoking status replaced with smoker, as per Russell standard)
(3a) Reduce incentive amount (max £160) and lower quit rate: Too *et al*. [14]	Cost of intervention £131 (SE = 13.265) Quit rates incentives arm 0.046 (SE = 0.005) and control arm 0.025 (SE = 0.003)	Too *et al*., based on £160 maximum amount of vouchers and applying 24‐week quit rate as proxy for late pregnancy
(3b) Increasing incentive amount (max £800) and improved quit rate: Lussier *et al*. [37]	Cost of intervention with £800 maximum vouchers £463 (SE = 47.4) Quit rate for incentive arm 0.34 (SE = 0.035)	Based on Lussier *et al*.’s paper £800 maximum incentives and quit rate of 34%
(4) Relapse rate in ESIP model replaced with CPIT III trial data	CPIT III 1‐year relapse rate based on 6‐month post‐partum and extrapolated 0.814 (SE = 0.079) Model original 1‐year post‐partum relapse rate 0.47 (SE = 0.046)	CPIT III trial data; 1‐year relapse rate in M. Jones’ model is taken from M. Jones’ systematic review, in this sensitivity analysis, it is replaced with CPIT III relapse rate for 6‐month post‐partum which is extrapolated to 1 year

Abbreviations: CPIT, Smoking Cessation in Pregnancy Incentives Trial; ESIP, Economics of Smoking in Pregnancy; NICE, National Institute of Health and Care Excellence; QALY, quality‐adjusted life‐years; SE, standard error.

#### Analysis

Probabilistic sensitivity analysis (PSA) was conducted to allow characterization of uncertainty in parameters. Base‐case results for the following scenarios are presented: mother (pregnancy and life‐time), infant (pregnancy, childhood and adulthood) and combined life‐time (mother and infant).

Six sensitivity analyses were pre‐specified to explore the effect of varying model input parameters on results: (1) gaming (a) using quit rate based on gaming in trial (separate arms) and (b) based on gaming in incentives arm; (2) self‐reported late‐pregnancy CPIT III quit rate; (3) varying incentive amount and possible impact upon quit rates based on (a) Too *et al*.’s findings [14], decreased incentive amount and lower quit rate and (b) based on Lussier *et al*.’s findings [37], increased incentive amount and improved quit rate; and (4) applying CPIT III 6‐month post‐partum quit rate, extrapolated to 1 year, replacing existing 1‐year relapse parameter in the ESIP model.

Results are presented as mean and incremental cost and outcome for each arm with 95% CI. ICERs are presented with a 95% CI and probability of cost‐effectiveness at £20 000 willingness‐to‐pay threshold (where appropriate).

## RESULTS

### Within‐trial analysis

Nine hundred and forty‐four participants were randomized. Three withdrew and asked that their data be removed; these participants were excluded from the economic evaluation in line with the primary outcome analysis. Nine hundred and forty‐one participants remained in the analysis, 471 in the financial incentive arm and 470 in the control arm.

#### Missing data

The amount of missing data was similar in each arm; total costs 12% in both arms, late‐pregnancy QALYs 21 and 25% for incentives and control, respectively, and post‐partum QALYs 38 and 40% for incentives and control, respectively (Table 3). Three hundred and thirty participants were missing either total costs or late‐pregnancy QALYs, leaving 611 complete cases.

**TABLE 3:** Missing data.

Variable	Incentives arm (*n* = 471) *n* (%)	Control arm (*n* = 470) *n* (%)	Total (*n* = 941) *n* (%)
Stop smoking services costs	43 (9%)	49 (10%)	92(10%)
Nicotine replacement therapy costs	56 (12%)	57 (12%)	113 (12%)
Total costs	56 (12%)	57 (12%)	113 (12%)
Quitters, late pregnancy	59 (13%)	39 (8%)	98 (10%)
Quitters, post‐partum	94 (20%)	92 (20%)	186 (20%)
Utilities, baseline	1 (0.2%)	1 (0.2%)	2 (0.002%)
Utilities, late pregnancy	101 (21%)	115 (24%)	216 (23%)
Utilities, post‐partum	133 (28%)	132 (28%)	265 (28%)
Quality‐adjusted life‐years, late pregnancy	101 (21%)	116 (25%)	217 (23%)
Quality‐adjusted life‐years, post‐partum	177 (38%)	186 (40%)	363 (39%)
Missing late‐pregnancy quality‐adjusted life‐years or costs	157 (33%)	173 (37%)	330 (35%)

#### Number of vouchers issued

Three hundred and thirty‐seven vouchers (71.4% participants in incentives arm) were issued for initial engagement with services and setting a quit date, 171 (36.2%) were issued at 4‐week quit stage, 138 (29.2%) at 12‐week quit stage and 150 (31.8%) at late pregnancy. Three vouchers were either stolen or sent and not received: one each for engagement and setting quit date, 4‐week quit stage and late‐pregnancy quit; these were excluded from the analysis. Three hundred and forty‐four (72.9%) participants in the incentives arm received one or more vouchers.

#### Base‐case analysis

The incentives arm was more costly than control in the short term; this difference was driven by neonatal costs (£1723 versus 982 incentives and control arms, respectively) (Table 4). Intervention costs are higher in the incentives arm compared to control (£268 versus 91), with £152 of those costs relating to vouchers.

**TABLE 4:** Short‐term costs breakdown (unadjusted).

	Incentives Mean (SE)	Control Mean (SE)
Issued vouchers and postage	£152 (7.63)	£0 (NA)
Stop smoking services	£52 (2.54)	£41 (2.30)
Nicotine replacement therapy	£64 (5.15)	£50 (4.43)
*Total intervention costs*	£268 (11.9)	£91 (6.40)
Neonatal	£1723 (548)	£982 (378)
Total unadjusted costs	£1991	£1073

Abbreviations: NA, not available; SE, standard error.

However, adjusted results found the difference in total costs to be £637 (95% CI = £−872 to 2160); while there was a trend for higher total costs in the incentives arm, the 95% CI straddled zero (Table 5). Late‐pregnancy quit rate was higher in the incentives arm compared to control arm (0.268 versus 0.123), a difference of 0.144 (95% CI = 0.094–0.194). The incremental cost per late‐pregnancy quitter was £4400. QALYs were slightly higher in the incentives arm compared to the control arm (0.339 versus 0.335), a difference of only 0.004, and a 95% CI which again crossed zero, (95% CI = −0.163 to 0.175). The incremental cost per QALY was £150 000, which would not be considered cost‐effective given the UK willingness‐to‐pay threshold of £20 000/QALY [13].

**TABLE 5:** Short‐term analysis results.

Analysis	Cost/effect	Incentives Mean (standard error)	Control Mean (standard error)	Incremental	Incremental cost‐effectiveness ratio
Base case (cost per late‐pregnancy quitter)	Cost	£1799 (21)	£1161 (14)	£637 (−£872 to 2160)	
	Quitter	0.268 (0.020)	0.123 (0.015)	0.144 (0.094 to 0.194)	£4400 per quitter
	QALY	0.339 (0.002)	0.334 (0.002)	0.004 (−0.143 to 0.150)	£150 000 per QALY
S1: including miscarriage *n* = 18/944	Cost	£1805 (153)	£1154 (98)	£652 (−£934 to 2245)	£151 000 per QALY
	QALY	0.339 (0.01)	0.335 (0.01)	0.004 (−0.138 to 0.156)	
S2: self‐reported quit, late‐pregnancy missing *n* = 58 replaced as smoker status	Cost	£1799 (21)	£1161 (14)	£637 (−£872 to 2160)	£3700 per quitter
	Quitter	0.359 (0.022)	0.185 (0.018)	0.174 (0.118 to 0.230)	
S3: gaming, 11% incentives and 20% controls	Cost	£1799 (21)	£1161 (14)	£637 (−£872 to 2160)	£4500 per quitter
	Quitter	0.239	0.098	0.141	
S4: gaming, more conservative estimate 11% in both arms	Cost	£1799 (21)	£1161 (14)	£637 (−£872 to 2160)	£4900 per quitter
	Quitter	0.239	0.109	0.130	
S5: post‐partum quitter	Cost	£1799 (21)	£1161 (14)	£637 (−£872 to 2160)	£24 000 per quitter
	Quitter	0.079 (0.01)	0.051 (0.01)	0.027 (−0.004 to 0.059)	
S6: post‐partum QALY	Cost	£1799 (21)	£1161 (14)	£637 (−£872 to 2160)	£106 000 per QALY
	QALY	0.405 (0.01)	0.399 (0.01)	0.006 (−0.126 to 0.141)	
S7: complete case (*n* = 611)	Cost	1821 (159)	1589 (139)	£232 (−1718 to 2102)	£40 000 per QALY
	QALY	0.339 (0.02)	0.333 (0.01)	0.006 (−0.171 to 0.199)	
S8: neonatal costs equal	Cost	£1622 (0)	£1445 (0)	£176 (£174 to 179)	
	Quitter	0.268 (0.020)	0.123 (0.015)	0.144 (0.094 to 0.194)	£4500 per quitter
	QALY	0.339 (0.002)	0.334 (0.002)	0.004 (−0.143 to 0.150)	£41 360 per QALY
S9: postage £6	Cost	£1804 (21)	£1161 (14)	£644 (−£748 to 2432)	
	Quitter	0.268 (0.020)	0.123 (0.015)	0.144 (0.094 to 0.194)	£4500 per quitter
	QALY	0.339 (0.002)	0.334 (0.002)	0.004 (−0.143 to 0.150)	£151 000 per QALY
S10: postage £0	Cost	£1793 (21)	£1162 (14)	£631 (−£722 to 2265)	
	Quitter	0.268 (0.020)	0.123 (0.015)	0.144 (0.094 to 0.194)	£4400 per quitter
	QALY	0.339 (0.002)	0.334 (0.002)	0.004 (−0.143 to 0.150)	£158 000 per QALY

Abbreviations: QALY, quality‐adjusted life‐year; S, sensitivity analysis; Generalised linear model (GLM) regression results.

**FIGURE 2:**
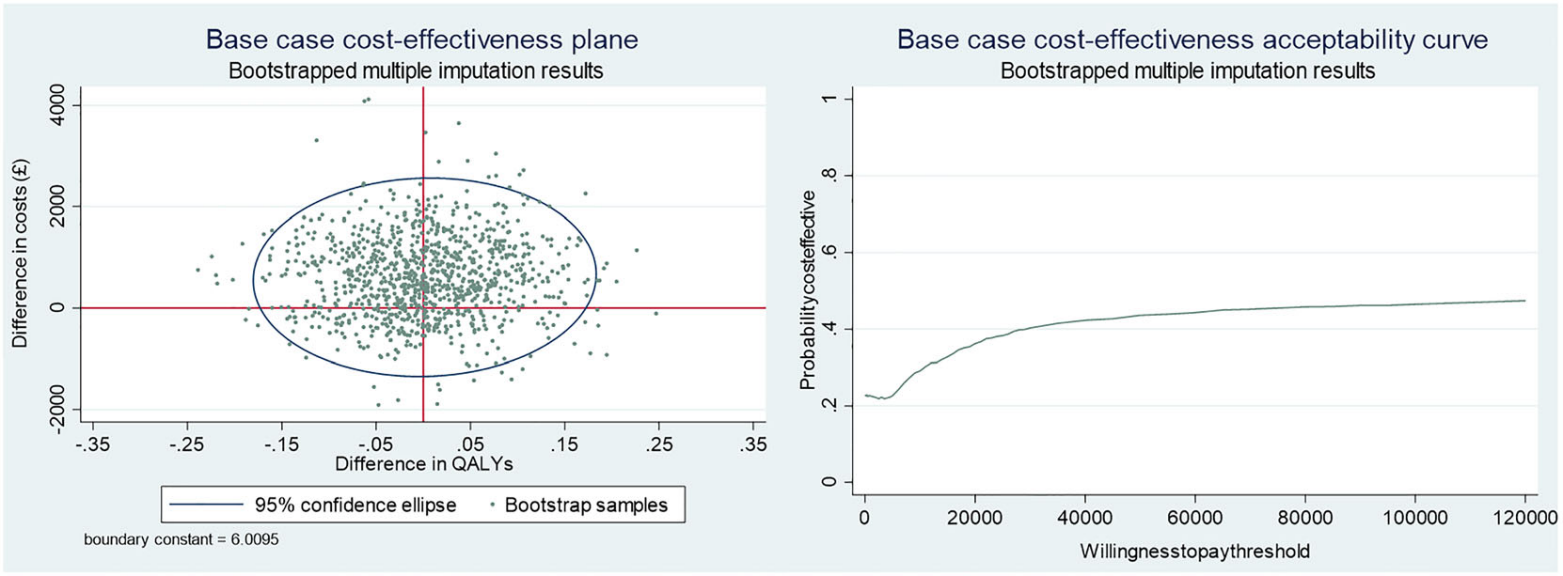
Base‐case cost‐effectiveness plane and cost‐effectiveness acceptability curve.

Uncertainty is illustrated on the cost‐effectiveness plane and CEAC (Figure 2). Bootstrapped samples cover all four quadrants of the cost‐effectiveness plane showing uncertainty in both cost and QALY results. There is less uncertainty in costs than QALYs; most samples indicate higher costs in the incentives arm compared to control (above horizontal axis). CEACs are used to explore the incremental cost‐effectiveness of an intervention at different willingness‐to‐pay thresholds [38, 39]. At the £20 000 willingness‐to‐pay threshold (the NICE accepted threshold [13]) the CEAC shows a 36% chance of incentives being cost‐effective in the short term, increasing to 40% at £30 000, and not rising above 47% up to £120 000 in the willingness‐to‐pay threshold. Therefore, offering financial incentives to this population is unlikely to be considered cost‐effective in the short term.

#### Sensitivity analyses

Results confirm base‐case results, with cost per quitter ranging from £3700 (self‐reported quit rate) to £24 000 (post‐partum quit). Cost per QALY results range from £40 000 (complete case) to £158 000 (£0 postage) (Table 5).

### Life‐time analysis

#### Base‐case results

Results show that incentives would be considered highly cost‐effective given the UK willingness‐to‐pay threshold of £20 000; for mother life‐time, infant end of childhood, infant adulthood and combined mother and infant (life‐time) scenarios (Table 6). For ‘maternal end of pregnancy’ scenario, the probability of being cost‐effective is 0%, with an incremental cost per QALY of £44 427. This is a similar conclusion to sensitivity analysis 8 in the short‐term results, and unsurprising given that the health benefits of quitting smoking are not immediate. Combined life‐time mother and infant results estimate cost savings of £37 (95% CI = −£35 to 106) and QALY gains of 0.171 (95% CI = 0.124–0.229). These results show that introducing financial incentives to usual care is a dominant strategy (cost‐saving and QALY‐gaining) using a life‐time horizon. The probability of being cost‐effective is 100%.

**TABLE 6:** Life‐time analysis results.

Analysis	Cost/effect	Incentives Mean	Control Mean	Incremental	ICER (95%CI)	Probability cost‐effective at £20 000 threshold
Base case (maternal, end of pregnancy)	Cost	£3392	£3213	£179 (£152 to 207)	£1242 (£1052 to 1436)	
	Quitter	26.8	12.3	14.4 (14.4 to 14.4)		
	QALY	0.691	0.687	0.004 (0.003 to 0.006)	£44 427 (£30 525 to 66 665)	0%
Base case (maternal lifetime)	Cost	£10 565	£10 472	£93 (£53 to 131)	£2964 (£1199 to 5946)	99.98%
	QALY	23.0	23.0	0.03 (0.018 to 0.056)		
Base case (infant, end of pregnancy)	Cost	£3493	£3328	£166 (£124 to 215)		NA
	Adverse live births	95	98	−4 (−4 to −3)	£44 743 (£31 412 to 61 412)	
	Adverse pregnancy outcomes	188	196	−8 (−9 to −7)	£20 003 (£14 447 to 26 677)	
Base case [infant, end of childhood (age 15)]	Cost	£5491	£5452	£39 (−£21 to 100)	£678 (−£364 to 1831)	100%
	QALY	10.2	10.2	0.06 (0.04 to 0.11)		
Base case (infant, adulthood)	Cost	£7899	£7858	£41 (−£19 to 103)	£306 (−£152 to 786)	100%
	QALY	23.8	23.7	0.136 (0.09 to 0.19)		
Base case [combined mother (life‐time) and infant (childhood and adulthood)]	Cost	£18 202	£18 239	−£37 (−£106 to 35)	Dominant	100%
	QALY	46.9	46.7	0.171 (0.124 to 0.229)		
S1a, Gaming	Cost	£18 274	£18 306	−£32 (−£98 to 37)	Dominant	100%
	QALY	46.8	46.7	0.167 (0.121 to 0.224)		
S1b, Gaming	Cost	£18 265	£18 280	−£16 (−£78 to 47)	Dominant	100%
	QALY	46.8	46.7	0.154 (0.112 to 0.207)		
S2, Self‐reported quit rate	Cost	£18 090	£18 169	−£79 (−£161 to 2)	Dominant	100%
	QALY	47.0	46.8	0.206 (0.149 to 0.274)		
S3a, Varying incentives	Cost	£18 370	£18 361	£10 (−£20 to 41)	£479 (−£1031 to 2110)	100%
	QALY	46.6	46.6	0.025 (0.018 to 0.033)		
S3b, Varying incentives	Cost	£18 298	£18 238	£60 (−£73 to 192)	£235 (−£297 to 779)	100%
	QALY	47.0	47.0	0.257 (0.188 to 0.345)		
S4, Varying relapse rates	Cost	£18 305	£18 272	£33 (−£35 to 100)	£225 (−£256 to 702)	100%
	QALY	46.8	46.7	0.146 (0.103 to 0.200)		

Abbreviations: ICER, incremental cost‐effectiveness ratios; NA, not available; QALY, quality‐adjusted life‐year.

**FIGURE 3:**
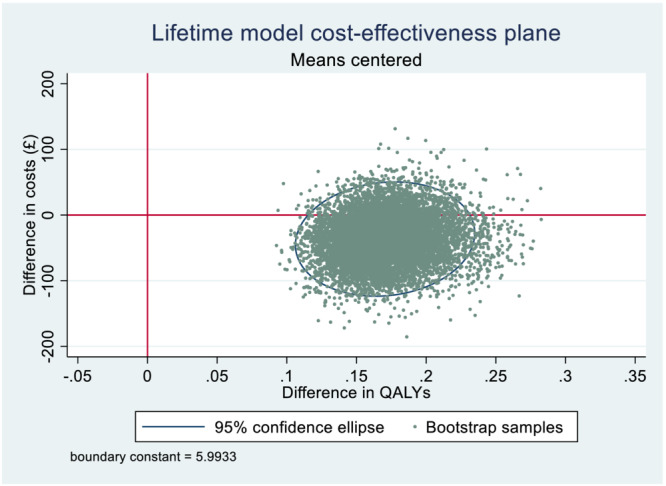
Life‐time model cost‐effectiveness plane.

Combined mother and infant (life‐time) results indicate little uncertainty (Figure 3). Ten thousand PSA samples show higher QALYs in the incentives arm compared to control in all samples and lower cost in most samples, where incentives are dominant (cost‐saving and QALY‐gaining).

#### Sensitivity analyses (mother and infant life‐time)

Results confirm base‐case long‐term results; financial incentives would be considered highly cost‐effective. Incremental cost per QALY ranges from a dominant strategy for self‐reported quit rate and both gaming scenarios to an ICER of £479 (95% CI = −£1031 to 2110) for using Too *et al*. [14].

## DISCUSSION

### Findings

Short‐term cost per quitter was £4400 and cost per QALY was £150 000. There was little uncertainty in difference in quit rates between arms; however, differences in costs and QALYs were inconclusive. As we would not anticipate short‐term quit rates to immediately translate into health gains, this result was expected, and the long‐term analysis results are more suitable to estimate cost‐effectiveness for decision‐making. The long‐term analysis found offering incentives to be cost‐saving (£37) with improved health benefits (quitters and QALYs). During a life‐time, offering financial incentives in addition to usual care is highly cost‐effective for mother and infant compared to usual care only. Sensitivity analyses for the short‐ and long‐term time horizons confirmed these results.

### Previous research

The CPIT II trial reported the short‐term cost per late‐pregnancy quitter of £1127 [12], lower than the present trial cost per quitter of £4400; this difference is largely due to the inclusion of neonatal costs in the present analysis. CPIT II did not report a short‐term cost per QALY, but reported a model‐based life‐time cost per QALY of £482. This is lower than the £2964 reported in the present study for a maternal life‐time cost per QALY. An evaluation of the implementation of maximum £160 financial incentives in NHSGG&C reported cost per quitter at 4 and 12 weeks of £517 and 546, respectively [14], again lower than the present study short‐term cost per quitter, but restricted to intervention costs and shorter time‐frame. A study assessing the cost‐effectiveness of up to $500 financial incentives for mothers receiving Medicaid reported a cost per 6‐month post‐partum quitter of $3399 [40]. Finally, a recent study assessing the cost‐effectiveness of offering maximum $1225 incentives reported an ICER of $23 511 per QALY at 24 weeks post‐partum [15]. However, neither of the latter two studies reported life‐time cost per quitter or QALY.

### Implementation

During the trial, three duplicate vouchers were issued and treated as a research cost. If financial incentives were implemented in a health‐care setting, it is likely that duplicate vouchers would occur at additional cost, as well as postage and staff time administering financial incentives. Scenario analyses explored alternative postage costs, with minimal impact on ICER results. In terms of implementation, it would be appropriate to consider alternative voucher types and distribution methods to improve efficiency, such as electronic vouchers or mobile phone app, which could be ‘topped up’ remotely or by way of codes when self‐reported quits are validated. These would be relatively cheap compared to the trial methods employed for voucher distribution.

### Strengths and limitations

Strengths of the economic evaluation include the trial being pragmatic, reflecting actual practice at seven sites across Scotland, Northern Ireland and England and providing evidence on the success of financial incentives in real‐world situations. Research shows maternal smoking during and after pregnancy can have serious negative consequences on the infant. The effects of maternal smoking were incorporated into our long‐term analysis to reflect the impact on mothers and infants. There is little evidence on quit rates and quality of life post‐partum; however, we collected these outcomes 6 months post‐partum; although these data were subject to missingness, it provides additional evidence in this field. Further, we input CPIT III post‐partum relapse rates into the ESIP model to reflect the rates witnessed in a trial; the resulting ICER showed that results would be considered cost‐effective.

Limitations of the economic evaluation include challenges during the COVID‐19 pandemic of collecting data. SSS support varied between sites, and data on individual NRT and SSS were limited to five of the seven sites. Costs for the two additional sites were based on data collected in the trial database, potentially reducing precision of our analyses. CPIT III trial did not collect neonatal stay data; therefore, preterm status and severity were used as a proxy indicator for neonatal stays. There was an imbalance in neonatal stays between arms, probably due to chance and the lack of precision of using a proxy indicator. As it is unlikely that quitting smoking increases neonatal cost, this is a potentially misleading finding which should be borne in mind when interpreting results. We carried out a sensitivity analysis (number 8) with neonatal costs equal between arms (based on the mean neonatal cost per person); the resulting ICER was very similar to the ‘end of pregnancy’ scenario for the long‐term model. While all relevant costs to the NHS were collected, patient expenses were not collected; these could include travel expenses, child‐care and informal support. Also, spill‐over effects were not included, such as partners quitting smoking.

There is sparse and variable evidence regarding whether the amount of financial incentives offered impacts the quit rate. Previous research suggests that increasing incentive amounts improves the effectiveness in substance use and smoking cessation [37, 41]. However, more recent research has shown no clear evidence of this link in health behaviour change [10, 42, 43]; indeed, with increasing incentive amounts, diminishing returns would set in due to a cubic trend [43]. Another barrier to estimating links between amount of incentive and magnitude of effect is that the success of financial incentives is also dependent upon the level of cessation support [44]; in CPIT III, cessation support was found to vary by site. Further research is needed into varying the amount of incentive and the resulting impact on effectiveness.

## CONCLUSIONS

Offering financial incentives alongside usual care is effective at improving quit rates in the short term and is highly cost‐effective for mother and infant over a life‐time. This research should prompt health‐care providers to offer financial incentives, alongside usual care, to pregnant women who smoke to encourage engagement with support service and improved quit rates.

## AUTHOR CONTRIBUTIONS

**Nicola McMeekin:** Formal analysis (lead); methodology (equal); writing—original draft (lead); writing—review and editing (equal). **Lesley Sinclair:** Data curation (equal); investigation (equal); project administration (equal); writing—review and editing (equal). **Lyn Robinson‐Smith:** Data curation (equal); investigation (equal); project administration (equal); writing—review and editing (equal). **Alex Mitchell:** Data curation (equal); methodology (equal); writing—review and editing (equal). **Linda Bauld:** Funding acquisition (equal); writing—review and editing (equal). **David Tappin:** Funding acquisition (lead); writing—review and editing (equal). **Kathleen Anne Boyd:** Conceptualization (lead); funding acquisition (equal); methodology (equal); supervision (lead); writing—review and editing (equal).

## DECLARATION OF INTERESTS

None.

### CLINICAL TRIAL REGISTRATION

Trial registration number: ISRCTN15236311, date registered 09/10/2017 https://doi.org/10.1186/ISRCTN15236311.

## Data Availability

The data that support the findings of this study are openly available and published by the Office for National Statistics: https://www.ons.gov.uk/peoplepopulationandcommunity/birthsdeathsandmarriages/deaths/adhocs/14989numberofdrugrelateddeathsbyindividualdayofoccurrenceenglandandwalesoccurredbetween1993and2018andregisteredbytheendof2021. The data are duplicated together with analysis code: https://github.com/danlewer/drd-time-trends. Ethics approval was received from NHS West of Scotland Research Ethics Committee, 15 August 2017.
